# Impact of minocycline on vascularization and visual function in an immature mouse model of ischemic retinopathy

**DOI:** 10.1038/s41598-017-07978-z

**Published:** 2017-08-08

**Authors:** Wenqin Xu, Jie Yin, Lijuan Sun, Zhicha Hu, Guorui Dou, Zifeng Zhang, Haiyan Wang, Changmei Guo, Yusheng Wang

**Affiliations:** 1 Department of Ophthalmology, Eye Institute of China PLA, Xijing Hospital, Fourth Military Medical University, Xi’an, China; 2 Department of Ophthalmology, Jinling Hospital, Nanjing, China

## Abstract

The role of microglia in the pathophysiology of ischemic retinal diseases has been extensively studied. Retinal microglial activation may be correlated with retinal neovascularization in oxygen-induced retinopathy (OIR), an animal model that has been widely used in retinopathy of prematurity (ROP) research. Minocycline is an antibiotic that decreases microglial activation following hyperoxic and hypoxic-ischemic phases in neonatal rodents. Here, we investigated the effects of minocycline on vascularization and visual function. In our results, we found that after the administration of minocycline, microglial reactivity was reduced in the retina, which was accompanied by an increase in the avascular area at P12, P14 and P17. Although microglial reactivity was reduced at P17, minocycline treatment did not attenuate retinal neovascularization. A changing trend in microglial number was observed, and the apoptosis and proliferation states on different days partly contributed to this change. Further study also revealed that although minocycline downregulated the levels of proinflammatory factors, visual function appeared to be significantly worsened. Collectively, we demonstrated that minocycline disturbed the physiological vascularization of the avascular area and exacerbated visual dysfunction, indicating that minocycline may not be an effective drug and may even be detrimental for the treatment of ischemic retinopathy in immature mammals.

## Introduction

Retinopathy of prematurity (ROP) is a retinal neurovascular disease that mainly affects prematurely born infants and has significant long-term effects on vision^1, 2^. Oxygen-induced retinopathy (OIR) in the mouse or rat, which produces reproducible and quantifiable proliferative retinal neovascularization, is a suitable model for examining the pathogenesis and therapeutic interventions of ROP^3, 4^. In the murine retina, physiological vascularization sprouts from the optic disc at birth and spreads to the periphery, which is reached at postnatal day 8 (P8)^5^. The state of retinal vascular development at murine birth is equivalent to that in infants born before term^6^. The superficial vascular plexus in the nerve fiber layer is established by P10, followed by the deep vascular plexus in the outer plexiform layer and the intermediate vascular plexus in the inner plexiform layer; this process is completed within approximately three weeks^5^.

In contrast to normal vascular development, vascular development is stalled in the OIR mouse model. During the hyperoxic period, a large avascular zone can be seen in the central retina at P12^7^; after mice are returned to normoxic conditions after P12, the central avascular area becomes hypoxic due to insufficient blood perfusion, which causes the upregulation of hypoxia-induced or -regulated growth factors^8–10^, leading to pathological retinal neovascularization and normal revascularization. After P17, retinal neovascularization tufts and clusters begin to regress, causing a morphologically normal retinal vascular system to develop^3^.

Microglia are activated in the mouse model of OIR during the hyperoxic period and the following hypoxic phase^11–13^. Moreover, strong evidence from animal models and human tissues has confirmed that microglia participate in retinal vascularization. Vessey *et al*. demonstrated that the increase in the number of microglia in mice with OIR correlated with the absence of the deep plexus^13^. One report demonstrated that an increase in microglial number may affect the onset of neovascularization^14^. Retinal microglia are derived from myeloid progenitor cells^15^ and are the immunological sensors of the retina. These active sensors of the microenvironment rapidly respond to various insults, transforming both morphologically and functionally into reactive phagocytes^16–19^.

Minocycline is a tetracycline antibiotic that can easily penetrate the central nervous system by crossing the blood-brain barrier after systemic administration; it has also been shown to alleviate the inflammatory response and provide neuroprotection in many models of brain and eye ischemic disease and injury. For instance, minocycline inhibits early diabetic retinopathy in the rat model by decreasing cellular apoptosis and caspase-3 activity in the retina^20^. In a study of retinal ischemia-reperfusion (IR) injury, minocycline treatment significantly inhibited IR-induced retinal vascular permeability and the disruption of tight junction organization but failed to block neurodegeneration^21^. As shown in brain research, minocycline significantly reduces infarct size and prevents tissue loss in the rat model of ischemic hemispheres, which is accompanied by improved perfusion^22^. In that study, the authors also found that treatment with minocycline significantly reduced the levels of tumor necrosis factor alpha (TNF-α) and interleukin 1 beta (IL-1β) and increased the levels of transforming growth factor beta (TGF-β), IL-10 and YM1. The administration of minocycline decreases microglial expression of chemotactic cytokines and reduces microglial infiltration and photoreceptor cell loss after subretinal hemorrhage *in vivo* ^23^. In these studies, minocycline has exhibited potential for therapeutic use. However, other results have shown that minocycline worsens injury-induced infarction and tissue atrophy in a mouse model of hypoxic-ischemia^24^. In conclusion, the effects of minocycline in the treatment of neurological ischemic diseases are contradictory.

The goals of our study were to understand the role of microglial activation in an OIR model and to test whether minocycline treatment can attenuate microglial activation together with retinal neovascularization and visual impairment. We assessed the effects of minocycline administration on vascularization status and microglial activation over several days in an OIR model in this study; our results describe dynamic changes in microglial activation and the effects of minocycline on microglial activation, apoptosis and proliferation. Inflammatory factors, including TNF-α, IL-1β, IL-4, IL-10, IL-12, inducible nitric oxide synthase (iNOS) and TGF-β, were detected, and electroretinograms (ERGs) were performed at P25 to evaluate visual function.

## Results

### Minocycline perturbed vascularization in OIR

To evaluate the effect of minocycline on vascularization, we used lectin staining.

**Figure 1:**
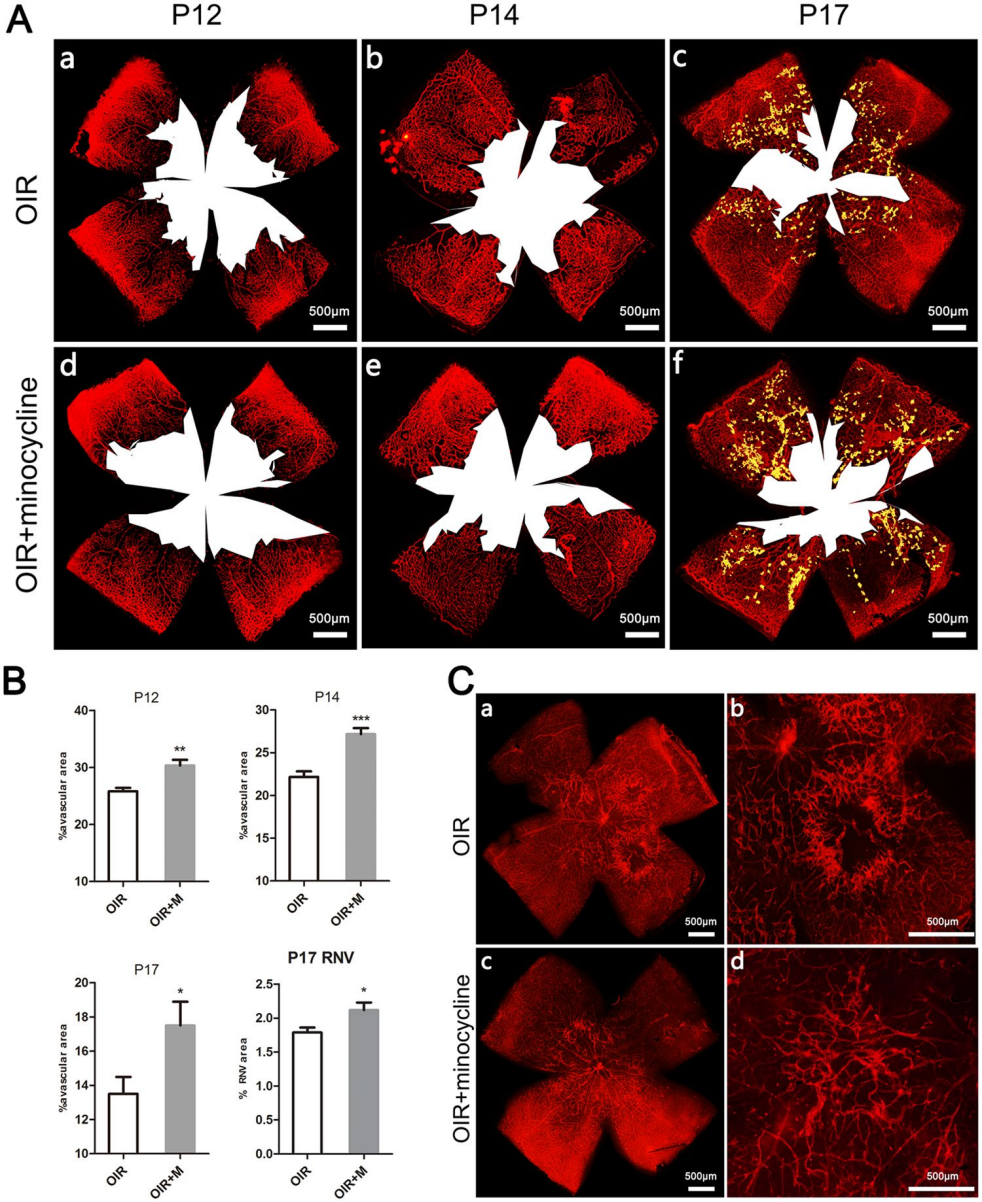
Minocycline increased the avascular area at P12, P14 and P17 and increased the neovascular area at P17. (**A**) The central avascular area was observed in the retina at P12 (a), P14 (b) and P17 (c) in the OIR group. Minocycline-treated mice exhibited an increase in the avascular area at P12 (d), P14 (e) and P17 (f). (**B**) The mean ratio of avascular area or neovascular area to total retinal area of the two groups at P12, P14 and P17. (**C**) Withdrawal of minocycline promoted vascularization of the avascular area. A small retinal avascular area was observed in OIR mice at P22 (a,b). Without minocycline administration for 6 days, the avascular area almost disappeared, even though some vessels exhibited abnormal morphology (c,d). OIR + M denotes the minocycline-treated group. Scale bar = 500 μm. Each column represents the mean ± SE (n = 6 per group), *P < 0.05, **P < 0.01, and ***P < 0.001.

In the OIR model, the retinas underwent vaso-obliteration during the hyperoxic phase and formed a central avascular area, which was notable at P12. When the mice were returned to normoxic conditions, new vascular tissue grew into the avascular area. We evaluated the central avascular area at P12, P14, and P17 and found that the percent of avascular area was higher in the minocycline-treated group from P12 to P17 (Fig. 1A and B). The retinal neovascularization state was compared at P17, and the administration of minocycline did not significantly attenuate neovascularization but rather increased it slightly (Fig. 1B). Interestingly, we observed that the hyperoxic minocycline-treated animal group accomplished vascularization by approximately P22, while the control group still had several avascular areas at the same time (Fig. 1C).

### The distribution of microglia and vessels

**Figure 2:**
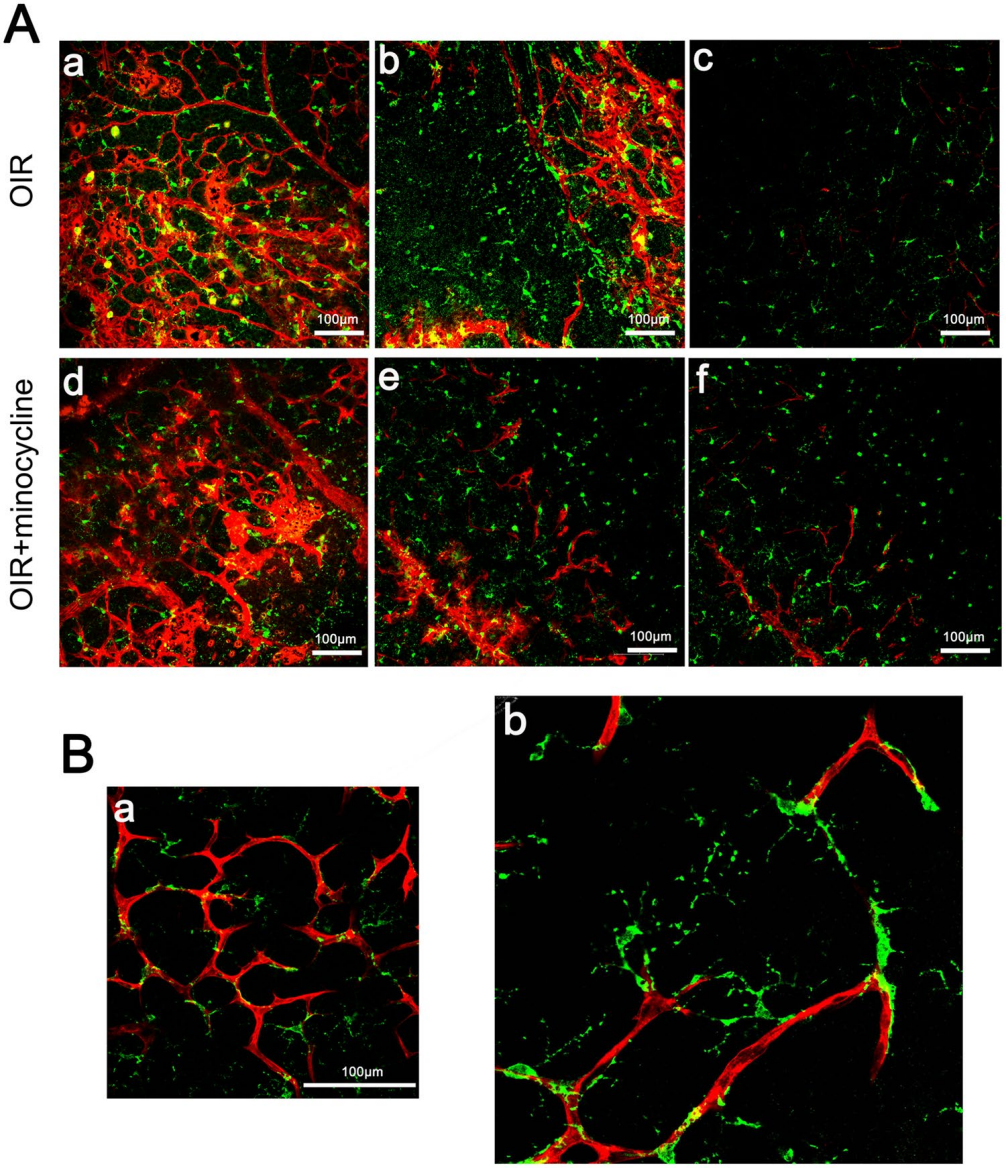
Distribution of microglia and vascular tissue in the retina at P17. Iba1 (green) and lectin (red) were used to stain microglia and blood vessels, respectively. (**A**) The neovascularization area (a,d), the border of the avascular area (b,e) and the deep layer (c,f) at P17 of the OIR and the minocycline-treated groups. (**B**) Synapses of microglia bridging vascular buds in retinas. Images were captured at 40X magnification (a) and 60X magnification (b). Scale bar = 100 μm.

Many microglia are assembled at neovascular tufts at P17, which we determined using retinal cryosections labeled with Iba1 immunofluorescence staining. Iba1-positive microglia are mainly found in the two following layers: the superficial layer, which is located in the retinal ganglion cell layer and the nerve fiber layer, and the deep layer, which is located at the border of the inner nuclear layer and the outer plexiform layer. We examined flat-mounted retinas labeled with Iba1 and lectin in our study. Aggregates of abundant activated microglia were observed over the neovascular tufts and border of the vascularized and avascular zone in the superficial layer at P17. In the deep layer, resting microglia were evenly distributed in the vascularized zone or assembled in the avascular zone, and a few activated microglia were found. Minocycline markedly decreased the density of activated microglia in the superficial layer; microglia mainly remained in the resting state in the deep layer (Fig. 2A). We also found that microglial synapses bridged the neovessel bud (Fig. 2B).

### Effect of minocycline on microglial activation

**Figure 3:**
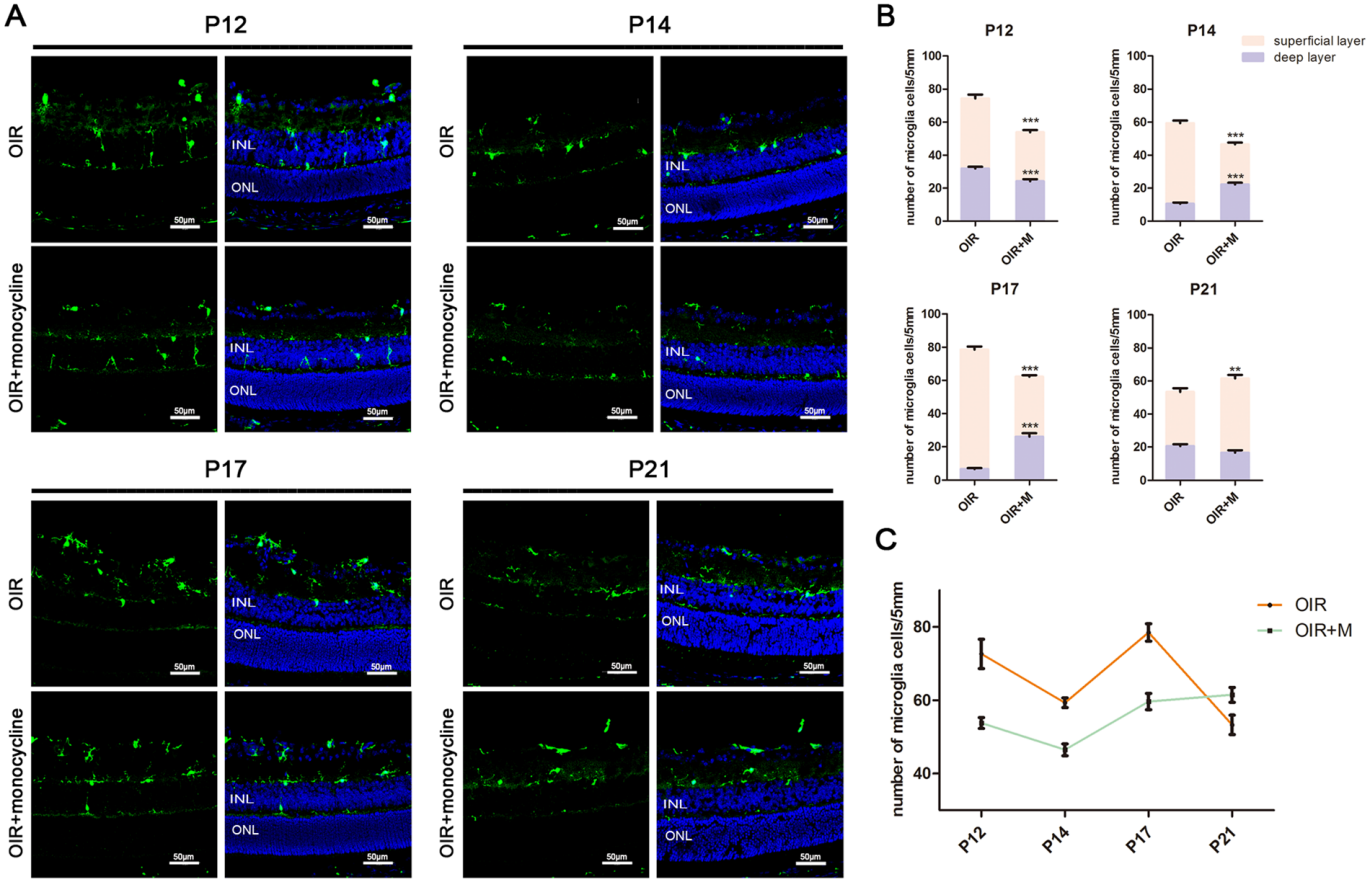
Cell densities of retinal microglia. (**A**) Iba1 (green) staining of the OIR and minocycline-treated groups at P12, P14, P17 and P21. (**B**) Microglial numbers in the superficial layer and deep layer on various days in the OIR and minocycline-treated groups. (**C**) The total Iba1-positive microglia per 5 mm of retinal length on different days. OIR + M denotes the minocycline-treated group. Scale bar = 50 μm, **P < 0.01, and ***P < 0.001 compared with the OIR group.

Iba1 positive microglia were scarce in cryosections of the normal eye from P12 to P21; however, numerous microglia were found in the OIR model retina, including the superficial and deep layers. During the hyperoxic phase, the microglial cell density continued to increase; however, after returning to normal room air, the number of microglia declined between P12 and P14. After reaching a minimum at P14, this number recovered gradually (Fig. 3A). Microglial density was always higher in the superficial than in the deep retinal layer. At P12, some microglia were present in the deep layer, and between P14 and P17, we barely detected any microglia; however, the microglia reappeared from P19 to P21 (Fig. 3B). We then examined the effect of minocycline on the inhibition of microglial activation. Minocycline was administered from P7 to P16, and injections of phosphate-buffered saline (PBS) were administered to control animals. Compared with the control animals, the minocycline-treated animals had fewer microglia in the retina from P12 to P17; however, after minocycline administration was halted, the number of microglia increased slightly in the hyperoxic minocycline-treated animal group (Fig. 3C).

### Apoptosis of microglia

**Figure 4:**
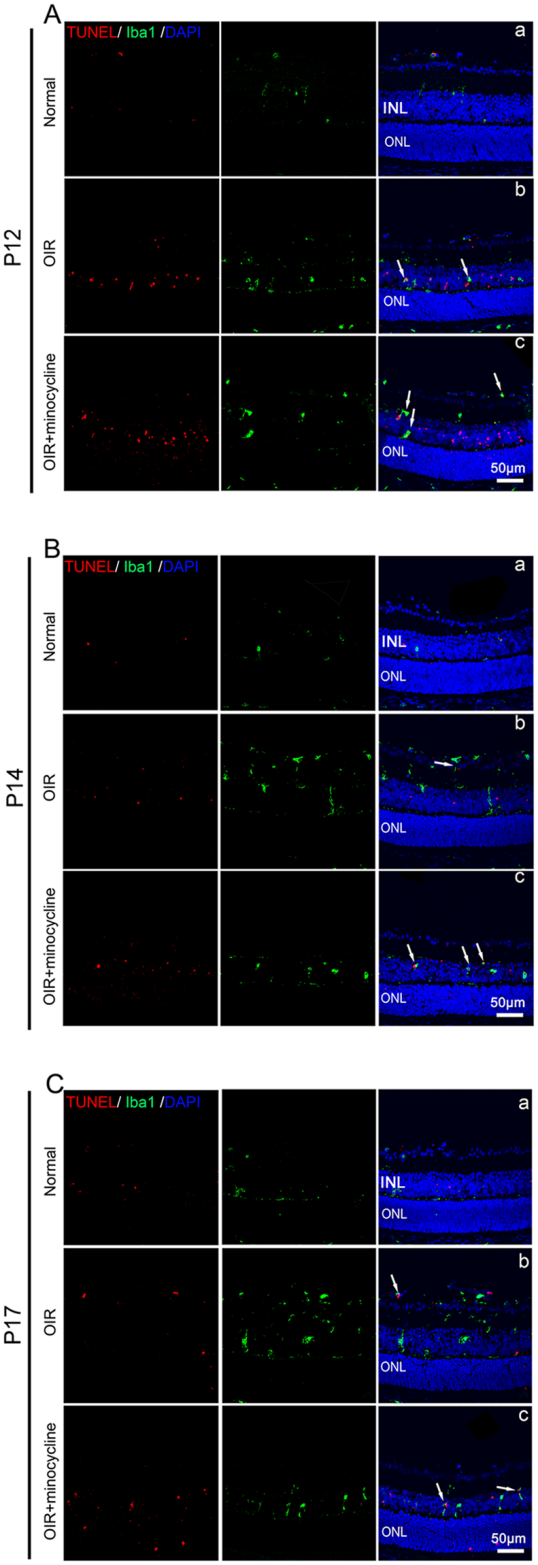
Apoptosis of microglia during different phases. (**A**) Representative images of Iba1 (green), TUNEL (red), and DAPI (blue) staining in the retinas of the normal (a), OIR (b) and minocycline-treated (c) mice at P12. (**B**) Representative images of the above three groups at P14. (**C**) Representative images of the above three groups at P17. Arrowheads point to double-positive cells, which are apoptotic microglia. Scale bars = 50 μm.

To determine whether minocycline affects microglial apoptosis, a TUNEL assay was performed. In the normoxic group, no apoptosis was observed in the retina, whereas in the OIR group, both hyperoxia and hypoxia caused apoptosis in the retina. Apoptosis was examined at P12, P14 and P17 (Fig. 4). Microglial apoptosis was most obvious on P12, when the mice were subjected to hyperoxic conditions. On P14, the number of microglia undergoing apoptosis declined. We demonstrated that the number of microglia decreased at P12 and P14. During this time, we found that apoptosis mainly occurred in the deep layer, which may partially explain this decline. At P17, microglial apoptosis was rare, and the microglia were mainly distributed in neovascular tufts. Surprisingly, we found that minocycline-treated animals exhibited more microglia undergoing apoptosis, which may partially explain why the retina exhibited fewer microglia.

### Proliferation of microglia

**Figure 5:**
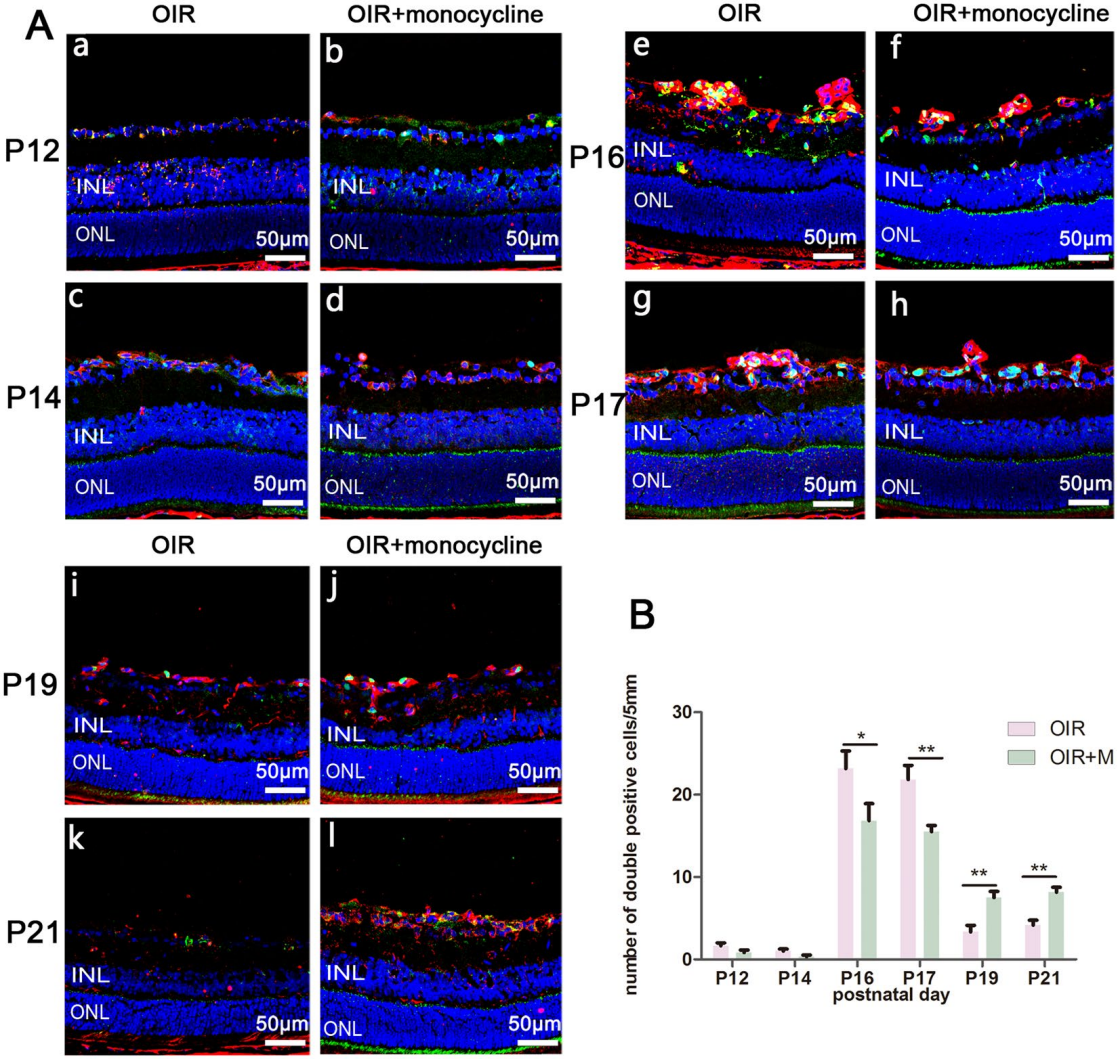
Cryosections showing proliferation in the OIR and minocycline-treated groups on different days. Ki67 staining (green) and CD11b (to label microglia, red) were used to detect proliferating microglia. (**A**) At P12 and P14, proliferating cells were rare in the OIR group (a,c), and microglia in the minocycline-treated group proliferated to a lesser extent (b,d). There were many more Ki67-positive cells at P16 and P17. Moreover, the number of double-positive cells in the OIR group (e,g) was higher than that in the minocycline-treated group (f,h). After the withdrawal of minocycline, retinas at P19 and P21 were harvested and analyzed. On P19, more microglia were proliferating in minocycline-treated mice (j) than in OIR mice (i). On P21, proliferation was not apparent in either group (k,l). (**B**) Quantification of double-positive cells in the retinas. OIR + M denotes the minocycline-treated group. Scale bar = 50 μm, *P < 0.05 and **P < 0.01.

The abovementioned results show that when the administration of minocycline was stopped, the density of microglia increased quickly, even exceeding the control group at P21. Accompanying this process, revascularization of the central avascular area was accelerated and was much faster than that in the control group. However, in the group of animals that were treated with minocycline from P17 to P21, revascularization was delayed as the activation of microglia was inhibited (data not shown). We wanted to understand why the microglial density presented a ‘catch-up and surpass’ phenomenon; therefore, the effects of minocycline on microglial proliferation were assessed *in vivo*. No significant difference was found in CD11+/Ki67+ numbers between the minocycline-treated group and the control group from P12 to P14 because proliferation was rare. However, on P16 and P17, we found numerous double-positive cells in the retina, mainly in the superficial layer and around neovascular tufts in OIR mice and minocycline-treated mice. In minocycline-treated animals, a more diffuse pattern of Ki67 staining was observed in the microglia, suggesting an inhibitory effect of minocycline on microglial proliferation. On P19 and P21, this state was reversed; as minocycline was withdrawn, more proliferating microglia were found in the minocycline-treated group than in the OIR group (Fig. 5). Thus, minocycline treatment inhibited microglial proliferation, and microglial proliferation increased as minocycline was withdrawn.

### Minocycline affected the expression of inflammatory factors

**Figure 6:**
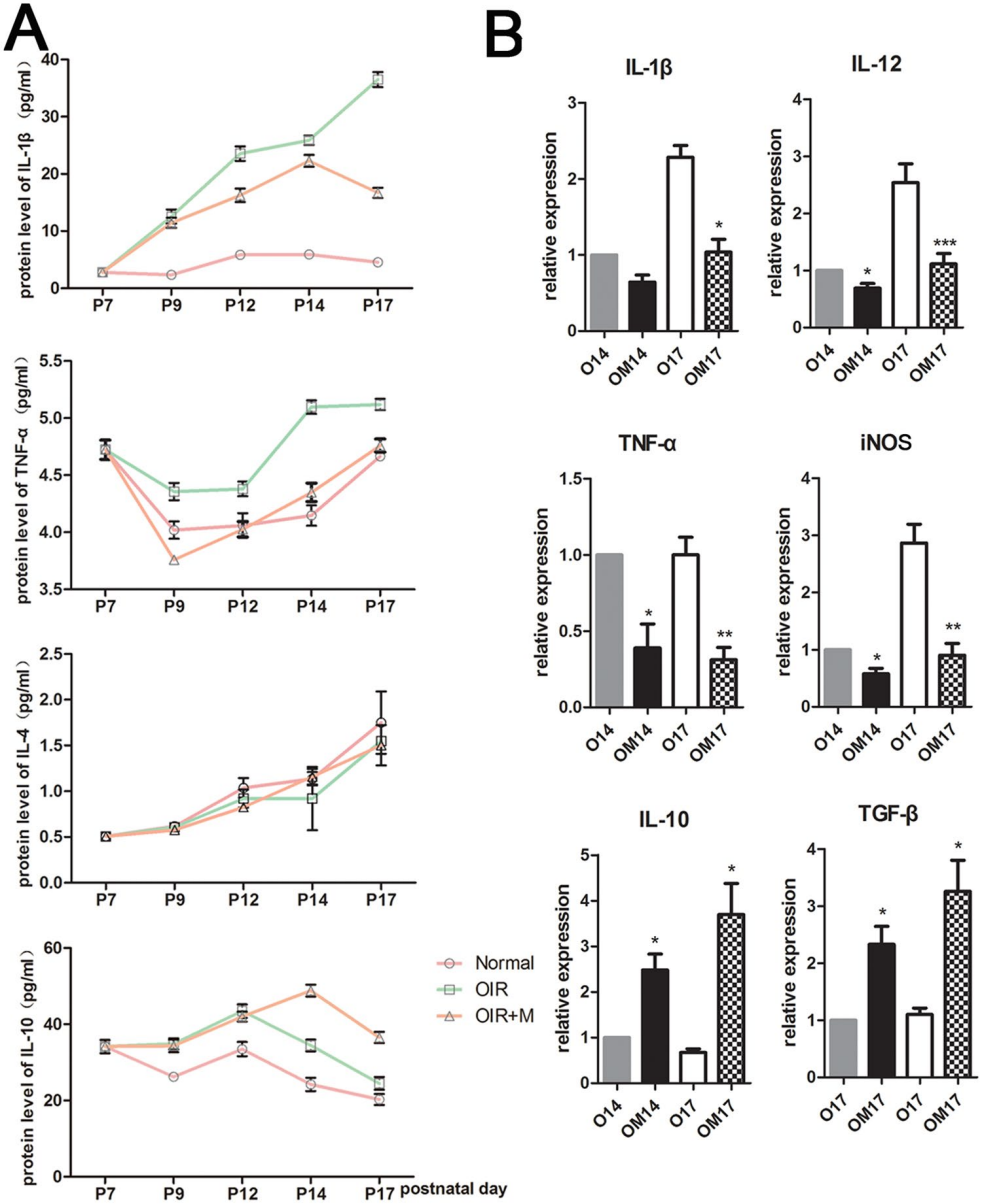
Expression of cytokines in the retina in the studied groups. (**A**) Concentrations of IL-1β, TNF-α, IL-4 and IL-10 in the retina of normal mice, OIR mice and minocycline-treated mice. OIR + M denotes the minocycline-treated group. (**B**) mRNA expression levels of IL-1β, IL-12, TNF-α, iNOS, IL-10 and TGF-β as determined by real-time RT-PCR in the retinas of the OIR and OIR + M groups at P14 and P17. O14 and OM14 denote the OIR group and the minocycline-treated group on P14, respectively. O17 and OM17 denote the OIR group and the minocycline-treated group on P17, respectively. All data are presented as the mean ± SEM, and n = 3–4 for all groups. *P < 0.05, **P < 0.01, and ***P < 0.001 compared with the OIR group.

Retinas were dissected from eyes on P7, P9, P12, P14 and P17, and retinal levels of the inflammatory cytokines IL-1β, TNF-α, IL-4 and IL-10 were measured using a Luminex system. The levels of IL-10, IL-1β and TNF-α were higher in the OIR group than in the normoxic group on P9, P12, P14 and P17. From P9 to P12, IL-10 levels generally increased, reaching 43.45 ± 3.06 pg/ml on P12, before decreasing during the hypoxic phase; on P14 and P17, the concentrations were 34.45 ± 2.67 pg/ml and 24.49 ± 2.89 pg/ml, respectively. Minocycline promoted the expression of IL-10 on P14 and P17, when the concentrations were 48.83 ± 2.69 pg/ml and 36.54 ± 2.56 pg/ml, respectively, and were higher than those in the OIR group. On P17, the concentrations of IL-1β and TNF-α reached their highest levels (16.70 ± 1.56 and 4.76 ± 0.10 pg/ml, respectively) in the OIR group, while minocycline reduced the expression of IL-1β and TNF-α. These results demonstrate that minocycline promoted the expression of IL-10 on P14 and P17 but reduced the expression of IL-1β and TNF-α (Fig. 6A). RT-qPCR was used to determine the RNA levels of IL-1β, IL-12, TNF-α, iNOS, IL-10 and TGF-β. Minocycline treatment inhibited IL-1β, IL-12, TNF-α and iNOS expression but increased IL-10 and TGF-β RNA levels at both P14 and P17 (Fig. 6B).

### Minocycline reduced visual function in OIR mice

**Figure 7:**
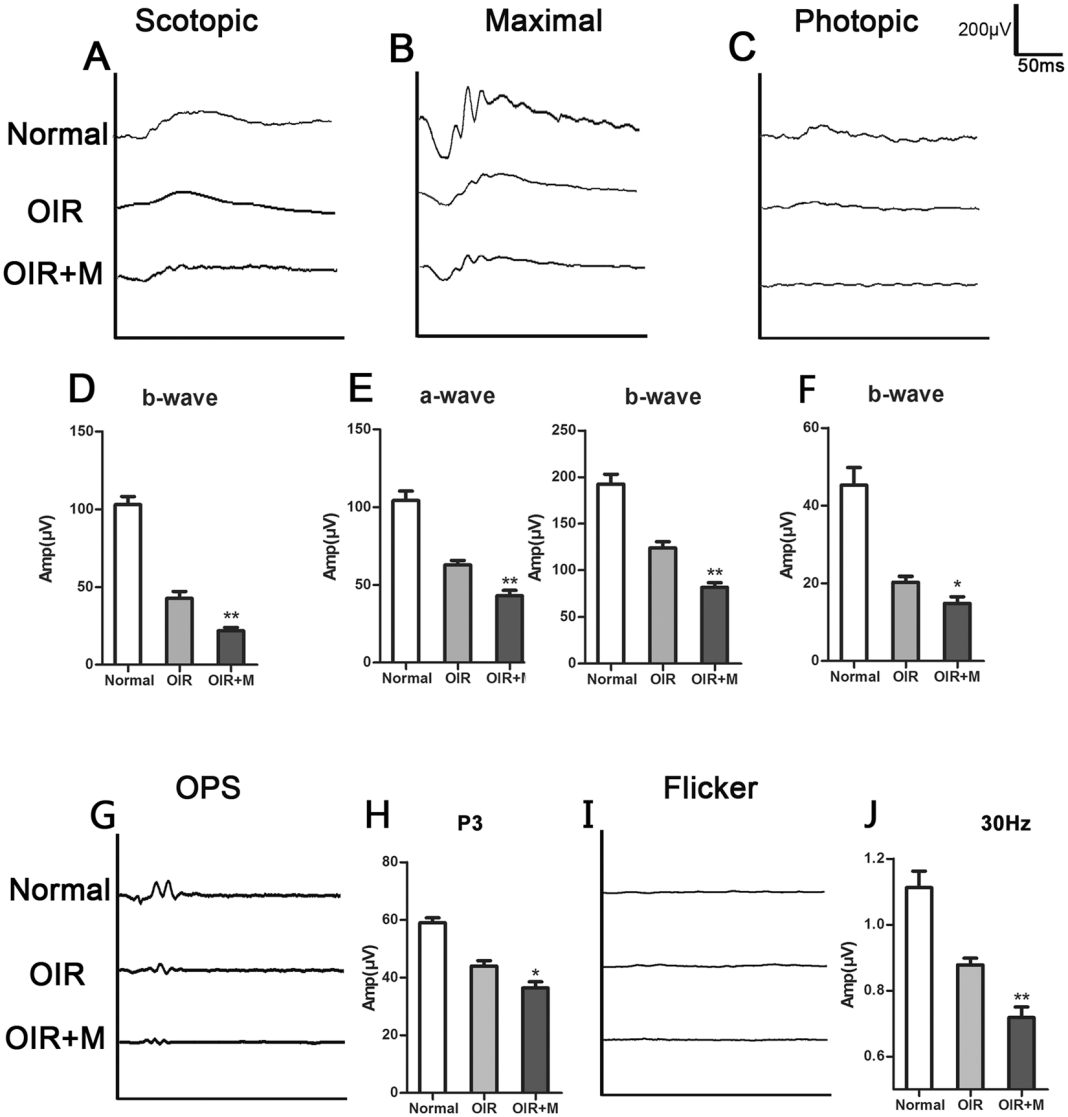
Visual function assessment by electroretinography (ERG). All mice underwent full dark adaption overnight before ERG. (**A**) Scotopic ERG measurement of normal, OIR and minocycline-treated mice at P25. (**B**) Maximal ERG response of the mice described above. (**C**) Photopic ERG measurements of the mice described above. (**G**) The maximal amplitude of OPs and the 30 Hz amplitude of Flicker. The wave graphs (**A**,**B**,**C**,**G**,**I**) indicate the average waveforms from each group, and the bar graphs (**D**,**E**,**F**,**H**,**J**) are the corresponding histograms of the wave amplitude values respectively. OIR + M denotes the minocycline-treated group. The data are presented as the means ± SD (**D**,**E**,**F**,**H**,**J**). n = 5–6 per group. *P < 0.05, **P < 0.01, compared with the OIR group.

Although retinal neovascularization tufts and clusters regressed, and the retinal vascular system appeared morphologically normal around P25 in OIR mice, the damage to visual function was irreversible. Thus, we examined visual function using ERG to determine whether minocycline attenuated visual damage. In this experiment, the b-wave amplitudes of the scotopic and photopic phases, the a- and b-wave of amplitude maxima, the maximal amplitude of OPs and the 30 Hz amplitude of Flicker were recorded and compared. Minocycline significantly reduced the amplitudes of these waves (Fig. 7), indicating that the hyperoxic environment to which the mice were exposed seriously damaged their visual function and that minocycline exacerbated this injury.

## Discussion

This study demonstrated that minocycline administration during the hyperoxic and hypoxic phases of OIR development reduced microglial activation when evaluated before and upon termination of treatment (from P9 to P17). The reduction was accompanied by a significantly larger avascular area and slightly severe neovascularization. Six days after withdrawing minocycline, the avascular area decreased; moreover, blood vessel morphology was more structured compared with the results obtained before P17. There were more microglia in minocycline-treated mice than in PBS-treated mice. Minocycline also decreased some inflammatory factors while increasing others. The administration of minocycline resulted in a definite deterioration of vision.

Some previous studies have shown that microglia accumulate in neovascular tufts^11^, and one research group reported that microglial activation, not microglial density, is correlated with retinal neovascularization during the hypoxic phase^14^. They also demonstrated a dynamic change in microglial cell density from P9 to P17, consistent with our results.

The function of microglia has been discussed for several years because they not only act as immune cells in the CNS and retina but they also participate in vascular formation. Microglia are first detected in the human retina at 10 weeks of gestation, before astrocyte invasion and the onset of vasculogenesis. These microglia extend their processes to orientate toward and contact blood vessels as they form. Furthermore, the depletion of resident retinal microglia by an intravitreal injection of clodronate liposomes impairs developmental vessel growth and density^25^.

When BALB/C mice (including pups and their mothers) are placed in a high-oxygen chamber (75 ± 2%) by P7 for 5 days (the same method for the C56BL/6J mouse model used in our research), the vascularization of the avascular area is very rapid because BALB/C mice present almost the same avascular area ratio as that for C56BL/6J mice at P12. However, on P17, most of these BALB/C mice have completed their retinal vascularization, and no significant retinal neovascularization appears. Therefore, Ritter *et al*.^12^ compared C56BL/6J mice and BALB/C mice and found that the density of retinal microglia significantly differs between these two strains. BALB/C mice present a much higher density. In our research, we used minocycline to inhibit the activation of microglia and found that vascularization was retarded in the avascular area, consistent with the reports mentioned above.

However, in our study, we also found that the vascularization of the avascular area was more rapid in the treatment groups than in the control group after the withdrawal of minocycline; during this period (P19 to P21), the number of microglia rebounded to a higher level than that seen in the control group. To explain this phenomenon, we examined the effects of minocycline on microglial proliferation and apoptosis. Microglial proliferation was significantly affected by minocycline during P12 to P17; however, on P19, three days after withdrawing minocycline, greater microglial proliferation was seen, suggesting that the increasing numbers of microglia in the minocycline-treated group may be due to the increase in microglial proliferation observed after P17. In addition, we found that minocycline also promoted microglial apoptosis from P12 to P16. These data collectively suggest that minocycline mainly affects the number of microglia in the retina through inhibiting their activation, although proliferation and apoptosis were also affected.

Minocycline inhibits the expression of CD68, cell surface markers of M1 polarized microglia, and inflammatory cytokines (IL-1β and TNF-a) both *in vitro* and in an animal model of amyotrophic lateral sclerosis. However, M2 markers, such as IL-4, IL-10, CD206, Ym-1 and CD206, are not affected^26^. Minocycline may also promote the M2 polarization of microglia and inhibit the M1 polarization of microglia *in vivo* ^27^. Zhou Y *et al*.^28^ used mannosylated clodronate liposomes to selectively deplete M2 macrophages and microglia and demonstrated that M2 rather than M1 macrophages play an important role in promoting retinal neovascularization, probably by producing secreted factors. In our study, we also found significant decreases in the protein and mRNA levels of IL-1β and TNF-α *in vivo* and in the mRNA expression of iNOS, IL-12, and an M1 polarized microglial marker at P14 and P17. Interestingly, we also found an increase in IL-10 protein expression in the retinas of the minocycline-treated group and increases in the mRNA levels of IL-10 and TGF-β. However, the protein and mRNA expression levels of IL-4 and Ym-1 (data not shown) were not influenced, probably because the expression levels of IL-4 and Ym-1 were extremely low. We hypothesize that microglial polarization might also play a role in neovascularization. This conjecture will be explored in our future research.

We conducted this study based on the effects of minocycline on microglia. To our knowledge, minocycline is widely used in research on CNS diseases, including Alzheimer’s disease, ALS, stroke, and trauma^29–34^. In the animal model of neonatal hypoxia-ischemia^35^, a reduction in activated microglia was accompanied by an attenuation of neurologic injury, which resulted in sustained improvements in cerebral atrophy or memory impairment^36^. Minocycline also exerts anti-inflammatory effects by reducing apoptosis and inhibiting the expression of matrix metalloproteinases after focal cerebral ischemia, as reported in animal models of stroke^37^. In these studies, minocycline was used to inhibit the activation of microglia and showed a protective role in these diseases, and most of the studied diseases occurred in adult humans or were examined in adult mice. In our study, we found that minocycline inhibited the vascularization process and can be regarded as a destructive agent. However, the association between microglial inhibition and the alleviation of degeneration in brain injury is unclear. A lack of microglia increases the severity and volume of injury caused by brain ischemia-reperfusion^38^. One study showed that minocycline successfully reduces microglial activation but does not affect brain trauma-induced neurogenesis in a closed-head injury model^39^. The mechanisms of different diseases and different animal models are discrepant; based on our study utilizing developing pups, we inferred that pup development may have affected the outcome. Nevertheless, future studies may examine a wider range of functional outcomes that may allow researchers to better understand the effects of minocycline administration in the developing body. In addition, future investigations should evaluate the effect of minocycline on microglial phenotypes and the role of cell type in this retinal neurovascular disease.

In conclusion, we demonstrated that minocycline treatment successfully decreased microglial activation but disturbed retinal vascularization during the hyperoxic and hypoxic phases; this treatment also impaired vision at P25, indicating a worse outcome. Minocycline downregulated harmful mediators such as IL-1β and TNFα and upregulated mediators such as IL-10 and TGF-β. Collectively, these data show that microglia may act as important components of vascular development and that, although a general suppression of microglial activation can decrease the levels of harmful inflammatory factors, minocycline did not alleviate ischemia-induced vision loss. Further work is warranted to elucidate the relationship between microglial activation and vascular pathologies in retinal ischemic disease.

## Methods

### Animals

All animal procedures adhered to the Association for Research in Vision and Ophthalmology Statement for the Use of Animals in Ophthalmic and Vision Research. The protocols were approved by the Institutional Animal Ethics Committee of the Fourth Military Medical University. Pregnant C57BL/6J mice (virgin females 8–10 weeks of age and 20 g to 25 g after mating) were purchased at an exact gestational age from the Animal Research Center at the Fourth Military Medical University. The mice were acclimated to the animal facility for at least 7 days prior to experimentation, and procedures that minimize animal stress were followed.

### Mouse model of OIR

The pups and their mothers were placed in a high-oxygen chamber (75 ± 2%) by P7 for 5 days and were provided access to a standard mouse diet and water. All animals were then returned to room air (normoxic) conditions.

### *In vivo* administration of minocycline

Experimental animals received intraperitoneal injections of minocycline (45 mg/kg per day dissolved in PBS; catalog No. M9511-1G; Sigma-Aldrich Corp.) from P7 to P16 or from day 17 to day 21. As controls, experimental animals were treated with an equal volume of PBS on the same schedule.

### Retinal flatmounts

At P12, P14, P17 and P22, mice were anesthetized with an i.p. injection of 30 ml/kg body weight of 1% sodium pentobarbital (Sigma, St Louis, MO, USA, P3761), and their left eyes were enucleated and fixed in 4% paraformaldehyde for 4 hours. The corneas and lenses were dissected, and the retinas were stripped of the retinal pigment epithelium under a microscope. The retinas were placed in 4% paraformaldehyde for 4 hours and then washed in 1X PBS, pH 7.4. The retinas were embedded in 1% Triton X-100 and 1% BSA at 4 °C for 12 hours and then washed three times in 1X PBS before being incubated in GSL-IB4 isolectin (1:100, Vector Labs, California, USA) at 4 °C overnight in the dark. The retinas were then washed three times in 1X PBS, and radial cuts were made in the periphery of the retinas to allow whole flat-mounting on glass slides.

### Quantification of the avascular area in flat-mounted retinas

Images of stained flat-mounted retinas were photographed under a fluorescence microscope system (BX51; Olympus, Tokyo, Japan). Retinal cross-section images were captured using a light microscope (FV1000; Olympus). Digitized whole retina images were reconstructed using Photoshop CS3 software (Adobe Systems, USA). For each retina, the avascular, neovascular, and total areas were manually outlined and quantified by a masked observer according to the protocol proposed by Connor KM *et al*.^40^. The ratio of avascular area to total retinal area was then calculated.

### Immunofluorescence staining

Retinal flatmounts. Retinas were dissected as described above and fixed in 4% paraformaldehyde for 4 hours. The retinas were then incubated with primary antibodies against Iba1 (1:500, Wako, Japan) in 1% Triton X-100 containing 1% bovine serum albumin overnight at 4 °C. After three washes in 1X PBS, the retinas were incubated with diluted fluorescein-labeled secondary antibody Alexa Fluor 488-conjugated goat anti-rabbit IgG (Invitrogen, Rockford, IL, USA) and GSL-IB4 isolectin (lectin, 1:100, Vector Labs) for 8 hours at 4 °C for localization and vascular staining. 4 mice were used for this staining.

Retinal cryosections. The right eyes without lens and vitreous humor were embedded in Tissue-Tek optimal cutting temperature compound (Sakura Finetek, USA) for cryosectioning, and 8-um serial sections were cut (CM1800; Leica Instruments, Germany). The slides were incubated with the following primary antibodies: rabbit anti-Iba1, mouse anti-CD11b (ab1211, Abcam, UK), and rabbit anti-Ki67 (ab15580, Abcam) in a humidified chamber overnight at 4 °C. The slides were then washed in 1X PBS and incubated for 3 hours at room temperature with the following secondary antibodies: Alexa Fluor 488-conjugated goat anti-rabbit IgG (Invitrogen), Alexa Fluor 488-conjugated goat anti-mouse IgG (Invitrogen), and Cy3-conjugated goat anti-rabbit IgG (Invitrogen); the sections were then washed in PBS and labeled with DAPI (Molecular Probes, Thermo Fisher Scientific Inc., USA). In total, 95 mice were used in this part and in the retinal flatmount part.

To label apoptotic microglia, retinal cryosections were labeled using the terminal dUTP-mediated TUNEL technique, which was performed according to the manufacturer’s protocol (*In Situ* Cell Death Detection Kit TMR red; Sigma-Aldrich Corp.). The slides were then incubated with Iba1, followed by secondary antibodies and DAPI.

### Confocal imaging and analysis

After immunohistochemical staining and/or TUNEL labeling, cryosections were imaged using confocal microscopy (ECLIPSE Ti, Nikon, Germany, Japan; or FluoView 1000, Olympus Corp., Japan). The numbers of cells expressing Iba1/CD11b and/or staining with Ki-67/TUNEL were determined in eyes from P12 to P21. The section passing through the optic nerve was selected, and the imaging field was centered on the bilateral sides of the retina; images were collected at different magnification.

### Microglia quantification

We measured the length of the scleral borders. All were longer than 5 mm; therefore, we counted the double-labeled cells, measured the length of each retinal slide, and calculated the number of microglia per 5-mm length of outer nuclear layer. All immunopositive cells were counted in three to six sections per animal.

### Multiplex cytokine assay

The mice were anesthetized, and their eyes were enucleated and placed in cell lysis buffer (Millipore Corporation, St. Charles, MO) containing a protease inhibitor mixture (Sigma-Aldrich Corp) for 20 min. After centrifugation at 15,000 × *g* for 15 min at 4 °C, the concentration of the supernatant was determined using a bicinchoninic acid protein assay kit (Beyotime, China). The concentrations of cytokines, including IL-1β, TNF-α, IL-4 and IL-10, were measured in duplicate using a Luminex system (Millipore Corporation., Germany) according to the manufacturer’s protocol. The results were normalized based on the protein concentration of the samples. In this part, 45 mice were used.

### Real-time PCR

The mice were anesthetized, and the eyes and retinas were quickly dissected and placed in sterile tubes. Total retina RNA was harvested using TRIzol reagent (Invitrogen, USA) according to the manufacturer’s instructions. Complementary DNA (cDNA) was synthesized using a cDNA Synthesis Kit (TaKaRa, Japan), and PCR amplification was conducted using a kit (SYBR Premix EX Taq, TaKaRa) and an ABI PRISM 7500 Real-time PCR system. Fourteen mice were used in this part. The primers used in the PCR are shown in the Table 1; β-actin served as a reference control.

**Table 1:** Primes used in PCR.

Name	Sequence
iNOS-F	5′-GCAGAGATTGGAGGCCTTGTG
iNOS-R	5′-GGGTTGTTGCTGAACTTCCAGTC
TNF-α-F	5′-CAGGAGGGAGAACAGAAACTCCA
TNF-α-R	5′-CCTGGTTGGCTGCTTGCTT
IL-1β-F	CCTGCAGCTGGAGAGTGTGGAT
IL-1β-R	5′-TGTGCTCTGCTTGTGAGGTGCT
IL-12-F	5′-GGAAGCACGGCAGCAGAATA
IL-12-R	5′-AACTTGAGGGAGAAGTAGGAATGG
IL-10-F	5′-CCCTTTGCTATGGTGTCCTT
IL-10-R	5′-TGGTTTCTCTTCCCAAGACC
TGF-β-F	5′-GACCGCAACAACGCCATCTA
TGF-β-R	5′-GGCGTATCAGTGGGGGTCAG
Actin-F	5′-CATCCGTAAAGACCTCTATGCCAAC
Actin-R	5′-ATGGAGCCACCGATCCACA

### ERG data

After overnight dark adaption, the mice were deeply anesthetized using an i.p. injection of 3 ml/kg body weight of 1% sodium pentobarbital (Sigma, St Louis, MO, USA, P3761), which was diluted with saline and 50 μl sumianxin II (Jilin Shengda Animal Pharmaceutical Co., Ltd., Jilin, China). The pupils were dilated with 0.5% tropicamide-phenylephrine ophthalmic solution (Shenyang Xingqi, Pharmaceutical Co., Ltd., Shenyang, China). The active electrode, a silver-chloride electrode loop encased in a layer of 1% methylcellulose, was applied to each cornea. The reference and ground electrodes were separately inserted beneath the skin of the cheek around the tested eye and tail. Full-field (Ganzfeld) stimulation and a commercial system (RETI port; Roland Consult GmbH, Brandenburg, Germany) were used to record ERGs with a bandpass of 0.5–1000 Hz. All operations were conducted under dim red light to maximize retinal sensitivity. Scotopic 0.01 cd.s.m-2, 3.0 cd.s.m-2 (maximal), 3.0 cd.s.m-2 OP ERG, photopic 3.0 cd.s.m-2, and 3.0 cd.s.m-2 flicker ERG were recorded. Fifty mice were used in this part.

### Statistical analysis

Statistical analysis was performed using GraphPad Prism 5. The data are presented as the means ± SD. Variance was similar between the compared experimental groups. Comparison between two independent means was performed using Student’s t-test. Differences between three groups were compared using one-way analysis of variance followed by Tukey’s post hoc test. Differences were considered significant at P < 0.05.
